# SENIEUR status of the originating cell donor negates certain ‘anti-immunosenescence’ effects of ebselen and N-acetyl cysteine in human T cell clone cultures

**DOI:** 10.1186/s12979-014-0017-5

**Published:** 2014-11-30

**Authors:** Shiva Marthandan, Robin Freeburn, Susanne Steinbrecht, Graham Pawelec, Yvonne Barnett

**Affiliations:** Jena Centre for Systems Biology of Ageing- JenAge, Jena, Germany; Leibniz Institute for Age Research, Fritz Lipmann Institute, Beutenbergstrasse 11, D-07745 Jena, Germany; School of Science, University of the West of Scotland, Paisley Campus, Paisley, Scotland PA1 2BE UK; Tübingen Ageing and Tumour Immunology Group, Center for Medical Research, University of Tübingen Clinical School, Waldhörnlestr. 22, D-72072 Tübingen, Germany; School of Science and Technology, College of Arts and Science, Nottingham Trent University, Clifton Lane, Nottingham, NG11 8NS England UK

**Keywords:** Immunosenescence, Ebselen, NAC, Proliferative capacity, Lifespan, SENIEUR, DNA damage, GSH:GSSG ratio, MAP kinases, JNK, p38, ERK, Total glutathione

## Abstract

**Background:**

Damage to T cells of the immune system by reactive oxygen species may result in altered cell function or cell death and thereby potentially impact upon the efficacy of a subsequent immune response. Here, we assess the impact of the antioxidants Ebselen and N-acetyl cysteine on a range of biological markers in human T cells derived from a SENIEUR status donor. In addition, the impact of these antioxidants on different MAP kinase pathways in T cells from donors of different ages was also examined.

**Methods:**

T cell clones were derived from healthy 26, 45 and SENIEUR status 80 year old people and the impact of titrated concentrations of Ebselen or N-acetyl cysteine on their proliferation and in vitro lifespan, GSH:GSSG ratio as well as levels of oxidative DNA damage and on MAP kinase signaling pathways was examined.

**Results:**

In this investigation neither Ebselen nor N-acetyl cysteine supplementation had any impact on the biological endpoints examined in the T cells derived from the SENIEUR status 80 year old donor. This is in contrast to the anti-immunosenescent effects of these antioxidants on T cells from donors of 26 or 45 years of age. The analysis of MAP kinases showed that pro-apoptotic pathways become activated in T cells with increasing in vitro age and that Ebselen or N-acetyl cysteine could decrease activation (phosphorylation) in T cells from 26 or 45 year old donors, but not from the SENIEUR status 80 year old donor.

**Conclusions:**

The results of this investigation demonstrate that the biological phenotype of SENIEUR status derived human T cells negates the anti-immunosenescence effects of Ebselen and also N-acetyl cysteine. The results highlight the importance of pre-antioxidant intervention evaluation to determine risk-benefit.

**Electronic supplementary material:**

The online version of this article (doi:10.1186/s12979-014-0017-5) contains supplementary material, which is available to authorized users.

## Introduction

T cells need to undergo rapid clonal expansion upon antigenic stimulation to produce an immune response. Any factor that interferes with the ability of T cells to clonally expand may impact on the effectiveness of an immune response with the potential to render it sub-optimal.

Damage to T cells from reactive oxygen species (ROS), from both extrinsic and intrinsic (including sites of inflammation) sources may result in altered T cell function or T cell death [1,2]. Mammals have evolved defence systems, e.g. antioxidants and DNA repair systems, to help defend against the harmful effects of ROS [3]. Nonetheless these defence systems are not perfect, and can become overwhelmed. In addition we have established that DNA repair capacity declines with age *in vivo* [4] and in CD4^+^ T cell clones (TCCs) cultured *in vitro* [5,6]. This lack of optimal performance at all times by the defence systems may result in an accumulation of DNA damage to critical levels within T cells, resulting in cell cycle arrest or even apoptosis [7], with the potential to impact adversely on the T cell mediated immune response.

Previous work from our group has provided evidence of an increase in the level of ROS-induced DNA damage with age in CD4^+^ TCCs cultured at 20% O_2_ [3,8-10] and an increase in DNA damage and mutation with age in human lymphocytes [11]. A more recent study demonstrated anti-immunosenescence effects of two antioxidants, 2-phenyl-1,2-benzisoselenazol-3 (2H)-one (Ebselen; [12]) or N-acetyl cysteine (NAC; [13]) on CD4^+^ TCCs derived from healthy 26 year old and 45 year old donors [10]. In this paper we now detail the impact of each of these two antioxidants on CD4^+^ TCCs derived from a healthy 80 year old donor (conforming to the SENIEUR protocol for healthily aged individuals; [14]). The SENIEUR protocol helps to ensure the rigorous selection of healthily aged individuals. Evidence from the literature suggests an age-associated compromise of T cell function [15]. An inverse relationship between replicative capacity and donor age of TCCs has previously been reported [16]. However, there are exceptions where a straightforward relationship between age and T cell function breaks down. T cells from very healthy elderly donors, including those selected using the SENIEUR protocol [14] is one exception. In these cases individuals have been shown to be able to raise an effective immune response, contributed to by adequate T cell function [17-19]. We were interested to examine whether the anti-immunosenescent effects of Ebselen or NAC, which we have previously reported, in TCCs from donors of 26 or 45 years of age [10] were also present when TCCs from a SENIEUR selected healthy aged donor were tested.

Ebselen is a lipid soluble seleno-organic compound having glutathione peroxidase like activity which enables them to scavenge hydroxyl radicals and peroxides using glutathione (GSH) as a substrate [20]. Furthermore, Ebselen has the capability to inhibit the release of apoptotic factor cytochrome c [21]. The antioxidant potential of Ebselen has been previously demonstrated in a number of other cell lines; HepG_2_ cells [20], human HL-60 [22] and PC-12 cells [23]. Their ability to scavenge intracellular ROS resulting in reduction of hydroxyl radical formation may have contributed to the antioxidant potential in TCCs derived from healthy 26 and 45 year old donors displayed by their impact on certain markers of T cell integrity and function [10].

In terms of NAC, the presence of acetylated form of the amino acid L-cysteine and sulfhydryl groups enables them to act as a precursor of GSH synthesis and neutralise free radicals respectively [24]. Glutamate and cysteine share the same transporter in the body and elevation in levels of extracellular glutamate competitively inhibit cysteine transport resulting in depletion of intracellular GSH synthesis. The ability of NAC to raise GSH levels due to its capacity to donate cysteine amino acid may also supplement its antioxidant potential [25]. Previous studies have revealed the ROS scavenging potential of NAC in HeLa cells [26] and HepG_2_ cells [20].

Although they can cause damage within living systems, ROS act as signals/mediators in a variety of cellular processes including; cell function, proliferation, differentiation, cell damage and death. ROS act as intracellular signalling molecules within T cells [27], and they can mediate their effects via several signalling molecules such as calcium, protein tyrosine kinases (PTKs), protein tyrosine phosphatases (PTPs), serine/threonine kinases and phospholipases. ROS have been revealed to control cell proliferation induced by lectin and have an established role in protein tyrosine phosphorylation and activation of JNK1 [28]. Mitogen activated protein (MAP) kinases, a prominent family of protein kinases, operate through several pathways including, extracellular signal regulated kinases (ERK), c-Jun N-terminal kinase (JNK) and p38 kinase. These pathways are involved in proliferation, differentiation and apoptosis [23,29,30]. In the novel study described in this paper, the impact of Ebselen or NAC on different MAP kinase pathways in human CD4^+^ TCCs derived from healthy 26, 45 and 80 year old donors has also been investigated in an attempt to understand the contributory factors to any alterations in the biological endpoints measured in the supplemented TCCs.

## Methods

### Culture of TCCs and determination of their proliferative capacity and lifespan

Clone 399-37 was derived from a healthy 80 year old donor (conforming to the SENIEUR protocol for healthily aged individuals; [14]), Clones 400-23 and 385-7 were derived from a healthy 26 and 45 year donor respectively. Three independently derived ([31] – general reference for deriving the TCCs) human CD4^+^ TCCs of each of the three donors were separately maintained in culture in 24 well plates (5 wells, 2 ml medium per well) containing serum-free medium, X-Vivo 10 (Bio Whittaker) at concentrations of 2-4 × 10^5^ cells per well, along with 2 × 10^5^ gamma-irradiated (80 Gy) RJK853 cells per well (EBV-transformed B-lymphoblastoid cell line with complete hprt deletion), as feeder cells. The clones were maintained at 37°C under conditions of 5% CO_2_ and 95% air atmosphere and supplemented with 400 U/ml recombinant IL-2 (Chiron, UK) on days 1 and 4 of the 7 days cycle. A viable cell count was performed on harvested cells using a Neubauer Counting Chamber, and a new culture cycle was set up with fresh medium and RJK853 feeder cells on day 7 [3,8]. The proliferative capacity and lifespan were determined similar to the protocol described previously [3,8,10]. The TCCs used in this study were kindly provided by the group of Professor Graham Pawelec.

### Ebselen or NAC supplementation of TCCs

Further to our previous study [10] we examined the impact of titrated concentrations of Ebselen (0, 10, 30, 60, 100 μM) or NAC (0, 1.25, 5, 7.5, 10 mM) in three pooled 399-37 (80 year old) TCCs, 385-7 (45 year old) TCCs and 400-23 (26 year old) TCCs respectively. n = 3 in each case.

### Determination of levels of oxidative damage to DNA in TCCs derived from a healthy 80 year old donor

The levels and types of DNA damage in TCCs, supplemented with or without antioxidants, at various time points throughout their lifespan were assessed using a modified alkaline comet assay [3,8,10].

### Quantitative determination of GSH:GSSG ratio and total glutathione level in TCCs derived from a healthy 80 year old donor

A GSH:GSSG ratio assay kit was used to determine the ratio of reduced glutathione (GSH) to oxidized glutathione (GSSG), and total glutathione levels [10].

### Assessing the impact of antioxidant supplementation on MAP kinase signalling pathways in TCCs derived from donors of all three ages using SDS-polyacrylamide gel electrophoresis (PAGE) and Western Blotting

Ebselen or NAC supplemented and non-supplemented clones were harvested at different stages of their lifespan (at different PDs). Cells were washed in 1×PBS (pH 7.4). To prepare samples for western blot, cells were counted in a Neubauer Counting Chamber and an appropriate amount of cells were re-suspended in loading buffer and incubated at 90°C for 10 minutes. The samples were stored at -20°C and later used for SDS-PAGE. Loading buffer consists of 4% SDS, 40% Glycerin, 50 mM Tris/HCL (pH 6.8), 50 mM Dithiothreitol (DTT) and bromophenol blue. Whole cell extracts were electrophoresed on SDS-PAGE and transferred to nitrocellulose membranes (Protran; Schleicher and Schuell). The membrane was blocked in 5% skimmed milk/TBS-T (0.5 M Tris Base, 9% NaCl, 0.5% Tween 20, pH 8.4; Tween 20 [Carl Roth]) and incubated with protein-specific primary antibodies followed by horseradish peroxidase–conjugated species-specific secondary antibodies (Jackson ImmunoResearch Laboratories, Inc.). Signals were detected using the ECL reagent (GE Healthcare) on imaging film (BioMax; Kodak). Western Blot for Anti-β- Actin was performed as the loading control.

### Quantification of phosphorylated and total MAP kinase protein expression levels in TCC samples derived from donors for all the three ages

For immunodetection, primary antibodies were used at the following dilutions: Phospho JNK (1:50; 9251), Phospho p38 (1:100; 9211), Phospho p44/p42 [ERK1/2] (1:500; 9101), Phospho c-Jun (1:50; 2361), SAPK / JNK (1:50; 9252), p38 (1:100; 9212), p44/p42 [ERK1/2] (1:600; 4695) and Anti-β- Actin (1:10,000; A5316). All antibodies except Anti-β-Actin were purchased from Cell Signaling Technology, Boston, USA. Anti-β-Actin was purchased from Sigma-Aldrich. Secondary antibodies, conjugated to horseradish peroxidase (Dako) were used at 1:10,000 dilution and blots developed using ECL detection system and radiographic film (GE Healthcare, Germany). After the film development, quantification of the signal intensities of the bands in the Western blots was carried out using the Metamorph software [32]. The signal intensities of the bands representing the levels of phosphorylated or total proteins were normalized to the reference band of Anti-β- Actin.

### Statistical analysis of the samples

The results were tested for significance using paired two-sample type 2 Student’s t-tests assuming equal variances; p values are presented as appropriate.

## Results

### Effects of Ebselen and NAC on intracellular redox status (GSH:GSSG ratio) and total glutathione levels in human TCCs *in vitro* derived from a healthy 80 year old donor

**Figure 1:**
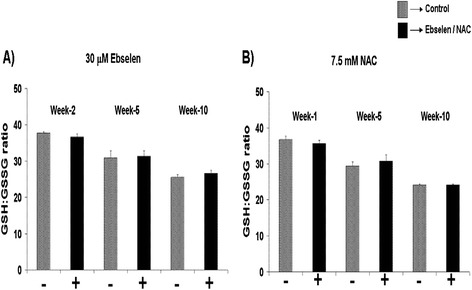
**Impact of 30μM Ebselen or 7.5mM NAC supplementation on GSH:GSSG ratio in TCCs derived from a healthy 80 year old donor. (A & B)** The impact of 30 μM Ebselen **(A)** or 7.5mM NAC **(B)** on GSH:GSSG ratio in three pooled TCCs derived from a healthy 80 year old donor. The bars indicate the mean ± S.D.

TCC samples were taken from the cultures at various time points during their lifespan and the effect of 30 μM Ebselen or 7.5 mM NAC on intracellular redox status (GSH:GSSG ratio) and total glutathione levels within the T cells were determined. Figure 1A and B show the results of the effect of 30 μM Ebselen or 7.5 mM NAC on the GSH:GSSG ratio.

Supplementation of TCCs from a young “*in vitro* age” with 30 μM Ebselen (Figure 1A) or 7.5 mM NAC (Figure 1B) had no impact on the GSH:GSSG ratio at any of the time points examined when compared to non-supplemented clones.

In both cases (antioxidant supplemented and non-supplemented), the GSH:GSSG ratio significantly decreased with increased time in culture (Additional file 1: Table S1A and B). A similar scenario was observed in TCCs derived from a healthy 26 and 45 year old donor in our previous study [10]. Furthermore, the GSH:GSSG ratio was significantly lower in TCCs derived from a healthy 80 year old donor (supplemented and non-supplemented) compared to the GSH:GSSG ratio in TCCs derived from either of the healthy young aged donors (26 and 45 year old; Tables 1 and 2; [10]).

**Table 1:** **GSH:GSSG ratio of TCCs + / - Ebselen**

**Times in culture (Weeks)**	**30μM ebselen**	**400-23 (26 yr ol)**	**385-7 (45 yr old)**	**399-37 (80 yr old)**
2	-	42.3 ± 1.7	44.3 ± 2.4	37.7 ± 0.4*
2	+	44.0 ± 1.7	41.9 ± 1.2	36.7 ± 0.9*
5	-	44.2 ± 2.8	42.0 ± 1.8	30.8 ± 2.0*
5	+	47.3 ± 2.4	46.8 ± 5.1	31.3 ± 1.5*
9/10	-	36.1 ± 1.6	33.4 ± 4.1	25.5 ± 0.7*
9/10	+	37.2 ± 1.4	36.3 ± 2.9	26.6 ± 0.8*

GSH:GSSG ratios in three pooled CD4^+^ TCCs (+ / - 30 μM Ebselen) derived from a healthy 80 year old donor are significantly lower than in either of the three pooled TCCs, each derived from a healthy 26 or a 45 year old donor (published data from [10]).

*Significantly lower GSH:GSSG ratio in 399-37 clones (80 year old) compared to either 400-23 (26 year old) or 385-7 (45 year old) clones.

**Table 2:** **GSH:GSSG ratio of TCCs + / - NAC**

**Time in culture (Weeks)**	**7.5 mM NAC**	**400-23 (26 yr old)**	**385-7 (45 yr old)**	**399-37 (80 yr old)**
2	-	46.3 ± 2.5	44.2 ± 0.8	36.7 ± 1.0*
2	+	51.2 ± 4.5	46.7 ± 3.3	35.7 ± 0.9*
5	-	37.9 ± 0.8	41.0 ± 0.5	29.4 ± 1.2*
5	+	42.5 ± 2.5	46.1 ± 0.3	30.7 ± 1.8*
9/10	-	35.5 ± 4.0	33.2 ± 3.1	24.2 ± 0.2*
9/10	+	35.8 ± 2.3	36.6 ± 2.6	24.1 ± 0.2*

GSH:GSSG ratios in three pooled CD4^+^ TCCs (+ / - 7.5 mM NAC) derived from a healthy 80 year old donor are significantly lower than the levels in either of the three pooled TCCs, each derived from a healthy 26 or a 45 year old donor (published data from [10]).

*Significantly lower GSH:GSSG ratio in 399-37 clones (80 year old) compared to either 400-23 (26 year old) or 385-7 (45 year old) clones.

Either concentration of Ebselen (30 μM) or NAC (7.5 mM) investigated in this study had any impact on total glutathione levels, at any of the three time points in TCCs derived from a healthy 80 year old donor compared to non-supplemented clones, as was the case for GSH:GSSG ratio. However, the levels of total glutathione were significantly lower in the 399-37 TCCs compared to levels in TCCs from both younger aged donors (Data not shown).

### The impact of Ebselen and NAC on *in vitro* proliferative capacity and lifespan of human TCCs derived from a healthy 80 year old donor

The TCCs used in this study underwent apoptosis at the end of their lifespan after completing a finite number of PDs. This is in line with previous reports [3,33].

The effect of different concentrations of Ebselen (0, 10, 30 μM) or NAC (0, 1.25, 5, 7.5 mM) on the proliferative capacity and *in vitro* lifespan of TCCs was investigated by supplementing them with one of the either antioxidants until the end of their lifespan. The results presented in Table 3 indicate that Ebselen (30 μM) and NAC (7.5 mM) supplementation of TCCs derived from a healthy 80 year old donor resulted in a slight decrease in the average number of PD accomplished per week, though not statistically significant. Neither of the antioxidants had any significant impact on the cumulative level of PDs achieved before the end of their lifespan in the TCCs derived from a healthy 80 year old donor, in contrast to the significantly enhanced PDs in antioxidant supplemented TCCs derived from the healthy younger donors ([10]; Table 3). However, 30 μM Ebselen or 7.5 mM NAC supplemented TCCs were able to survive in culture for an additional week and three weeks respectively compared to non-supplemented TCCs. Other concentrations investigated in the study, 10 μM Ebselen and 1.25 or 5 mM NAC did not reveal an impact on either proliferative capacity or lifespan in TCCs derived from a healthy 80 year old donor. Higher concentrations of Ebselen (60-100 μM) or NAC (10 mM) used in this investigation completely inhibited the growth of TCCs derived from a healthy 80 year old donor within a week of culture (Data not shown). A similar scenario was observed in our previous study when TCCs derived from a healthy 26 and 45 year old donor was supplemented with high concentrations of either Ebselen (60-100 μM) or NAC (10 mM), [10]. Furthermore, as explained in our previous study [10], mechanisms behind the pro-apoptotic effect of high concentrations of antioxidants have also been demonstrated in other model systems [34-36].

**Tables 3:** **Proliferative capacity and lifespan of TCCs on antioxidant supplementation**

**Clone (Age of the donor)**	**Initial PD**	**Concentration**	**Average PD per week**	**Cumulative PD achieved at the end of lifespan in culture**
400-23 (26 year old donor)	34.5	Control	0.7 ± 0.1	44.7 ± 0.3*
		30 μM Ebselen	1.2 ± 0.2	56.2 ± 0.4*
385-7 (45 year old donor)	31.0	Control	1.0 ± 0.1	44.4 ± 0.3*
		30 μM Ebselen	1.4 ± 0.4	50.8 ± 0.4*
399-37 (80 year old donor)	31.1	Control	1.2 ± 0.2	45.0 ± 0.4
		30 μM Ebselen	1.2 ± 0.1	44.6 ± 0.3
**Clone (Age of the donor)**	**Initial PD**	**Concentration**	**Average PD per week**	**Cumulative Pd achieved at the end of lifespan in culture**
400-23 (26 year old donor)	34.5	Control	0.7 ± 0.1	44.7 ± 0.3*
		7.5 mMNAC	1.2 ± 0.2	56.1 ± 0.3*
385-7 (45 year old donor)	31.0	Control	1.0 ± 0.1	44.4 ± 0.3*
		7.5 mMNAC	1.4 ± 0.4	55.8 ± 0.4*
399-37 (80 year old donor)	31.1	Control	1.1 ± 0.2	44.2 ± 0.5
		7.5 mMNAC	0.9 ± 0.2	42.3 ± 0.4

Effect of Ebselen or NAC supplementation on the proliferative capacity and lifespan of CD4^+^ TCCs derived from healthy 26, 45 or 80 year old donors.

Data of 26 and 45 year old donors are published data from [10]. *Significantly higher cumulative PDs in supplemented (+) clones compared to controls (-). n = 3 pooled TCCs for each age group.

### The impact of ebselen or NAC on levels of oxidative DNA damage in human TCCs as a function of *in vitro* age

Aliquots of TCC samples were taken from culture at various time points and the effect of 30 μM of Ebselen or 7.5 mM of NAC on the levels of oxidative DNA damage within the T cells were determined. In control (non-supplemented) samples, levels of oxidative damage to DNA increased as a function of age, as measured by the modified endonuclease III (Endo III) and formamidopyrimidine DNA glycosylase (FPG) comet assays, in line with previously published findings [3,9].

**Figure 2:**
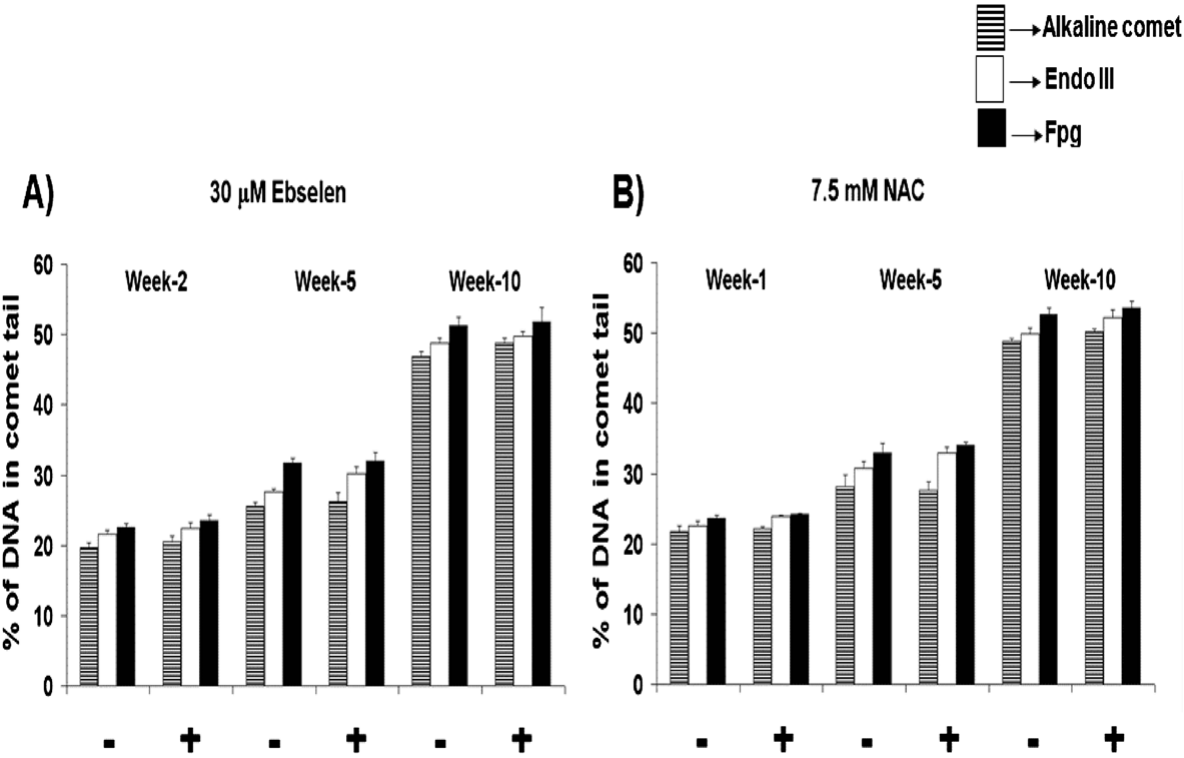
**Effect of 30μM Ebselen or 7.5mM NAC supplementation on the levels of oxidative DNA damage in TCCs derived from a healthy 80 year old donor. (A & B)** The impact of 30 μM Ebselen **(A)** or 7.5mM NAC **(B)** on the levels of oxidative DNA damage in three pooled TCCs derived from a healthy 80 year old donor. The bars indicate the mean ± S.D.

The results presented in Figure 2A and B reveal that oxidative DNA damage levels increased as a function of time in culture, in both supplemented and non-supplemented clones. Neither dose of antioxidants including 30 μM for Ebselen and 7.5 mM for NAC had any impact on the levels of oxidative DNA damage in TCCs during their span in culture. Figure 2 summarises the data obtained following 30 μM Ebselen (A) or 7.5 mM NAC (B) supplementation.

The levels of oxidative DNA damage significantly increased with time in culture in both supplemented and non-supplemented TCCs derived from a healthy 80 year old donor (Table 4). This was also the case in TCCs derived from a healthy 26 or 45 year old donor [10]. A comparison of the levels of oxidative DNA damage in the 399-37 clone samples with those from the younger donors revealed that basal levels of oxidative DNA damage were significantly higher (+/- supplementation) after all sampled time points (Additional file 1: Table S2A and B).

**Tables 4:** **Levels of oxidative DNA damage in TCCs on antioxidant supplementation**

**Time in culture (Weeks)**	**30 μM Ebselen (% of DNA in comet tail)**					
	-			+		
	**Alk**	**End**	**Fpg**	**Alk**	**End**	**Fpg**
2	19.7 ± 0.6	21.6 ± 0.6	22.5 ± 0.6	20.6 ± 0.8	22.5 ± 0.8	23.5 ± 0.8
5	25.6 ± 0.6	27.6 ± 0.4	31.7 ± 0.8	26.3 ± 1.2	30.1 ± 1.0	32.0 ± 1.1
10	46.9 ± 0.6	48.8 ± 0.7	51.2 ± 1.2	48.8 ± 0.7	49.8 ± 0.6	51.8 ± 2.1
	*	*	*	*	*	*
**Time in culture (Weeks)**	**7.5 mM NAC (% of DNA in comet tail)**					
	-			+		
	**Alk**	**End**	**Fpg**	**Alk**	**End**	**Fpg**
2	21.8 ± 0.8	22.6 ± 0.6	23.7 ± 0.3	22.2 ± 0.3	23.9 ± 0.1	24.2 ± 0.2
5	28.2 ± 1.6	30.8 ± 0.8	33.0 ± 1.2	27.7 ± 1.2	32.9 ± 0.8	34.1 ± 0.3
10	48.7 ± 0.4	49.9 ± 0.7	52.6 ± 1.0	50.2 ± 0.3	52.2 ± 1.2	53.6 ± 0.9
	*	*	*	*	*	*

The increase in levels of oxidative DNA damage in CD4^+^ TCC derived from a healthy 80 year old donor supplemented + / - 30 μM Ebselen or 7.5 mM NAC.

*Significantly higher levels of oxidative DNA damage in week 10 on comparison with the earlier weeks (2 and 5). n = 3 pooled TCCs for each age group.

### The impact of ebselen or NAC on different MAP kinase pathways in human TCCs derived from donors of different ages

The impact of Ebselen or NAC supplementation on MAP kinase phosphorylation status and total protein levels was determined in TCC samples from healthy young (26 year old), middle aged (45 year old) and elderly (80 year old) donors.

**Figure 3:**
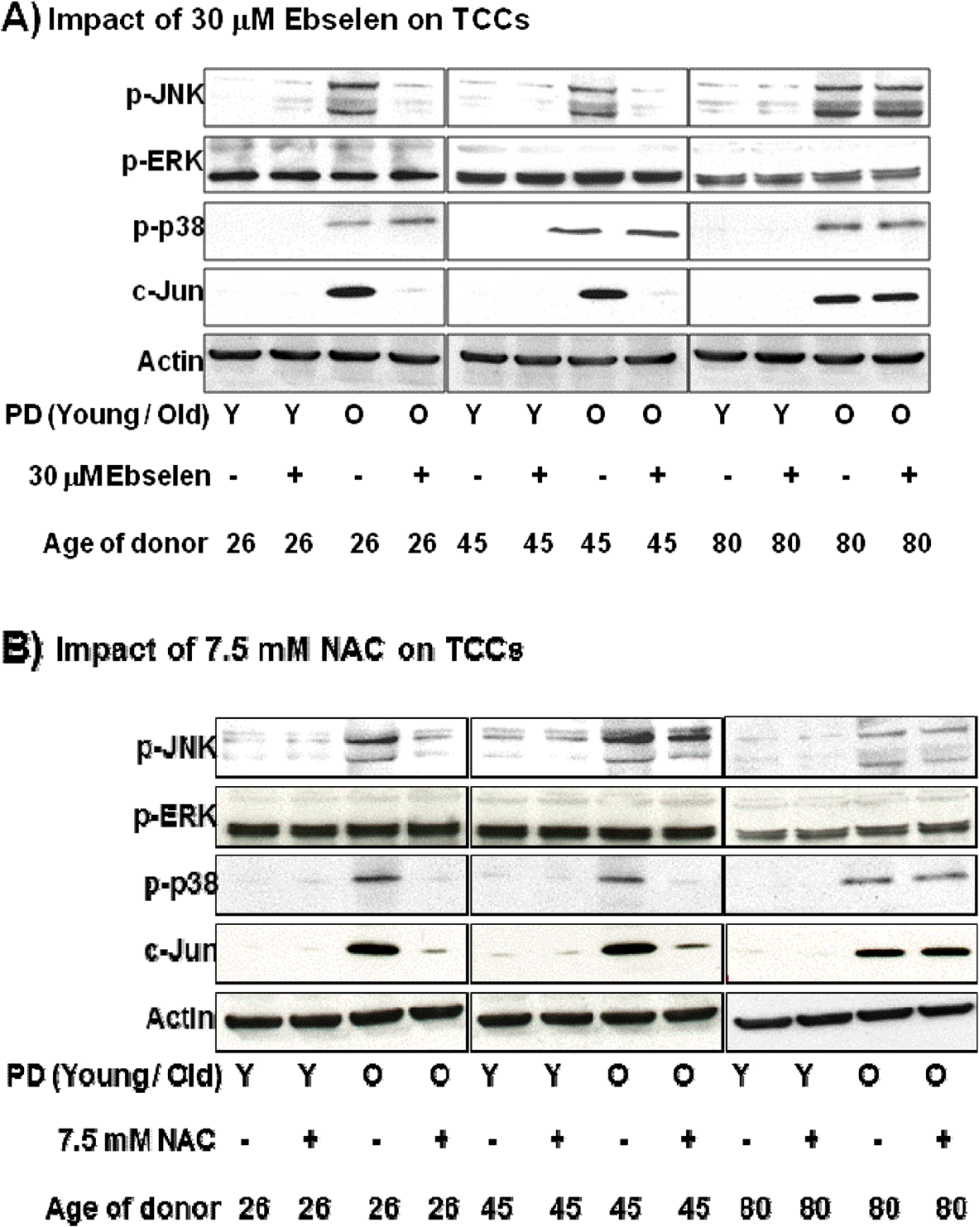
**Impact of 30 μM Ebselen (A) or 7.5 mM NAC (B) on the phosphorylation levels of JNK, c-Jun, p38 and ERK.** The blots reveal the effect of antioxidant supplementation between the young (early PD) and aged (late PD) TCCs isolated from healthy 26, 45 or 80 year old donors compared to non-supplemented controls.

Figure 3A reveals that ERK was similarly phosphorylated in Ebselen supplemented or control TCCs, irrespective of TCC *in vitro* age (PD). In contrast, JNK, p38 and c-Jun phosphorylation levels were absent (or low) in young cells (Y) but greatly enhanced in late PD cells (O) from all donors. 30 μM Ebselen did not significantly alter the increase of p38 phosphorylation in late PD TCCs. There was a significant reduction in JNK and c-Jun phosphorylation in young and middle-aged donor TCCs on Ebselen supplementation. However, Ebselen did not result in a reduction in JNK or c-Jun phosphorylation in TCCs derived from a healthy 80 year old donor (80, O, +). Quantification of the signal intensities of the bands in the Western blots were performed for both supplemented and non-supplemented clones (Additional file 1: Figure S1A-L).

A similar pattern of phosphorylation is seen in the young (early PD) TCCs with and without 7.5 mM NAC supplementation, with only ERK phosphorylated to any significant extent (Figure 3B). Phosphorylation of JNK, p38 and c-Jun was absent (or low) in young cells (Y) but greatly enhanced in aged cells (O) from all donors. 7.5 mM NAC supplementation inhibited this phosphorylation by at least 80% (Figure 3B) in young (26 year old) and middle aged (45 year old) donor TCCs with the exception of p-JNK in the middle aged donor TCC where a lower reduction was seen (~25%). However, no significant reduction in phosphorylation of JNK, p38, and c-Jun was found in TCCs derived from a healthy 80 year old donor treated with 7.5 mM NAC (80, O, +). Quantification of the signal intensities of the bands in the Western blots were performed for both supplemented and non-supplemented clones (Additional file 1: Figure S2A-L).

The total levels of JNK, p38 and ERK (Additional file 1: Figure S3C and D) were not significantly different following 30 μM Ebselen or 7.5 mM NAC, compared to non-supplemented controls.

## Discussion

Previous work from our group demonstrated the anti-immunosenescence potential of certain concentrations of Ebselen (30 μM) or NAC (7.5 mM) in CD4^+^ T cells ex vivo and in CD4^+^ TCCs when supplemented from a young *in vitro* age [10]. The ROS scavenging potential of these antioxidants resulted in enhancement of the GSH:GSSG ratio, a significant decrease in levels of oxidative DNA damage and a significant increase in lifespan, and/or proliferative capacity of TCCs derived from a healthy 26 year old or 45 year old donor.

In contrast, in the present study, supplementation of a TCC derived from a healthy 80 year old donor (conforming to the SENIEUR protocol; [14]) with 30 μM Ebselen or 7.5 mM NAC, from a young *in vitro* age (31.1 Initial PD) did not significantly alter the lifespan, the proliferative capacity (Table 3), levels of oxidative DNA damage (Figure 2A and B), intracellular redox status (GSH:GSSG ratio; Figure 1A and B) or the total glutathione levels.

Barnett and colleagues have previously published that 20 mM Carnosine (an antioxidant) supplementation from the midpoint of their *in vitro* lifespan did not alter the longevity of TCCs derived from an 80 year old donor [3]. In that case, it was suggested that Carnosine may not have been able to reveal its antioxidant potential due to the high background of biomolecule damage that already existed in these T cells, accumulated during earlier stages of their *in vitro* lifespan under conditions of 20% O_2_ that may have compromised a range of intracellular systems. One piece of evidence in this regard is the measured increase in basal levels of oxidative DNA in TCCs from a healthy 80 year old donor, compared with basal levels in TCCs from healthy 26 or 45 year old donors (Additional file 1: Table S2A, B). The results obtained in this current investigation suggest that a range of antioxidant supplementations do not have an impact on the biological endpoints measured in the TCCs from healthy 80 year old donors.

A similar scenario is applicable in terms of the GSH:GSSG ratio. Intracellular redox status (as reflected in the GSH:GSSG ratio) is an important mechanism having an invaluable role as a mediator in apoptosis in many cell systems [37]. Previous findings reveal that intracellular reduced glutathione (GSH), a main determinant of intracellular redox status, is depleted before the onset of apoptosis [38]. The GSH:GSSG redox couplet maintains the redox environment of the cell and GSH is abundant in the cell [39]. The oxidation of even a small amount of GSH results in the formation of GSSG thereby lowering the GSH:GSSG ratio suggested to be responsible for several human diseases [40]. However, in this study, the GSH:GSSG ratio did not significantly change on antioxidant supplementation compared to the non-treated controls, and the ratio decreased as cells approached the latter stage of their *in vitro* lifespan.

Although ROS are generally thought of as harmful molecules, they do play an important role in T cell signalling events [41] including the MAP kinase pathways. MAP kinases have several pathways identified including ERK, JNK and the p38 kinase pathways. ERK phosphorylation has been shown to act as a cell survival factor against oxidative stress, whereas phosphorylation of JNK and p38 contributes to cell death machinery [23]. T cell signalling events such as protein tyrosine phosphorylation and activation of JNK as well as cellular proliferation induced by lectin are some of the few instances that require the presence of ROS [28]. Reduced ROS levels might interfere with the signalling pathways involved in T cell activation and proliferation, for example, the redox-sensitive activation of transcription factors such as nuclear factor kappa light chain enhancer of activated B cells (NF-kB) or activator protein-1 (AP-1) [42].

This paper describes the investigation of the effect of the antioxidants, Ebselen or NAC, on the phosphorylation of p38 and JNK (SAPK) in TCCs from donors of different ages. JNK activation primarily results in apoptosis by the phosphorylation of c-Jun (serine 63), which is a component of the transcription factor complex AP-1 that binds to a specific DNA sequence in the AP-1 site [43] resulting in increase of DNA binding and ultimately apoptosis. Previous findings have indicated inhibition of H_2_O_2_-induced p38 MAP kinase activation, c-Jun phosphorylation and JNK activation by Ebselen in a concentration-dependent manner [23]. Furthermore, earlier studies have revealed that NAC decreased both JNK and p38 phosphorylation induced by 2,3,5-tris-(glutathion-S-yl)hydroquinone (TGHQ) in human epithelial cells [44], selenite in hepatocytes [29] and taxol (chemotherapeutic agent) in leukaemic cells [30]. The results of the present study suggest that pro-apoptotic pathways become activated in all TCCs as the cells reach an ‘old’ *in vitro* age with activation of JNK, p38 and c-Jun across all old TCCs irrespective of donor age (Figure 3A and B). The results of our study also reinforce the radical scavenging potential of Ebselen and NAC with a significant decrease in phosphorylation of JNK and c-Jun in late PD TCCs *in vitro* derived from a healthy 26 or 45 year old donor on supplementation with 30 μM Ebselen (Figure 3A) or 7.5 mM NAC (Figure 3B) compared to non-supplemented TCCs, although only NAC supplementation was able to decrease p38 phosphorylation in these late PD TCCs. However, neither antioxidant could significantly alter phosphorylation of p38, JNK or c-Jun in late PD TCCs *in vitro* derived from a healthy 80 year old donor (Figure 3A and B). Our results suggest that neither Ebselen nor NAC could alter the activation of p38, JNK and c-Jun in TCCs from very healthy elderly donors and thus fail to impact upon the time to onset of apoptosis. This is another piece of evidence which suggests that there are alterations to intracellular processes, which have accumulated during the prolonged existence of T cells from elderly donors.

In contrast to the results obtained from our study, others have published that NAC supplementation increased ERK activation in human kidney proximal tubule epithelial cells (HK-2) [44]. However, results from this present study revealed consistent activation of ERK in all TCCs irrespective of donor or *in vitro* age with no significant change in the levels of ERK phosphorylation in any of the age groups upon supplementation with either 30 μM Ebselen or 7.5 mM NAC, compared to non-supplemented TCCs (Figure 3A and B).

The results of this study highlight a heterogeneous potential of Ebselen or NAC as anti-immunosenescent interventive strategies in human T cells. If *in vivo* antioxidant supplementation is to be attempted then careful pre-intervention evaluation should be undertaken to determine risk-benefit.

## Additional file

Additional file 1: Table S1. Decrease in GSH:GSSG ratio levels with time in culture of 80 year old TCCs. **Table S2.** (A) Higher levels of oxidative DNA damage in 80 year old TCCs + / - Ebselen**. Table S2.** (B) Higher levels of oxidative DNA damage in 80 year old TCCs + / - NAC. **Figure S1.** (A-D) Effect of Ebselen on MAP kinase pathways in 26 year old TCCs. **Figure S1.** (E-H) Effect of Ebselen on MAP kinase pathways in 45 year old TCCs. **Figure S1.** (I-L) Effect of Ebselen on MAP kinase pathways in 80 year old TCCs. **Figure S2.** (A-D) Effect of NAC on MAP kinase pathways in 26 year old TCCs. **Figure S2.** (E-H) Effect of NAC on MAP kinase pathways in 45 year old TCCs. **Figure S2.** (I-L) Effect of NAC on MAP kinase pathways in 80 year old TCCs. **Figure S3.** (C) Impact of 30 μM Ebselen (C) on total levels of JNK, c-Jun, p38 and ERK. **Figure S3.** (D) Impact of 7.5 mM NAC (D) on total levels of JNK, c-Jun, p38 and ERK.

## Abbreviations

AP-1: Activator protein-1
CD: Cluster of differentiation
ERK: Extracellular signal regulated kinase
GSH: Reduced glutathione
GSSG: Oxidised glutathione
HK-2: Human kidney proximal tubule epithelial cells
JNK: c-Jun N-terminal kinase
ERK: Extracellular signal regulated kinases
MAP: Mitogen activated protein
NAC: N-Acetyl cysteine
PD: Population doubling
TCCs: T cell clones
TGHQ: 2,3,5-tris-(glutathion-S-yl)hydroquinone
PTKs: Protein tyrosine kinases
PTPs: Protein tyrosine phosphatases
Endo III: Endonuclease III
FPG: Formamidopyrimidine DNA glycosylase

## References

[1] Barnett YA, Brennan LA, O’Farrell F, Hannigan BM (1995). Oxidant-induced stress response in lymphoid cells. Biochem Mol Biol Int.

[2] Naik AK, Tandan SK, Dudhgaonkar SP, Jadhav SH, Kataria M, Prakash VR, Kumar D (2006). Role of oxidative stress in pathophysiology of peripheral neuropathy and modulation by NAC in rats. Eur J Pain.

[3] Hyland P, Duggan O, Hipkiss A, Barnett C, Barnett Y (2000). The effects of carnosine on oxidative DNA damage levels and *in vitro* lifespan in human peripheral blood derived CD4^+^ TCCs. Mech Ageing Dev.

[4] King CM, Bristow-Craig HE, Gillespie ES, Barnett Y (1997). *In vivo* antioxidant status, DNA damage, mutation and DNA repair capacity in cultured lymphocytes from healthy 75–80 year old humans. Mutat Res.

[5] Annett K, Hyland P, Duggan O, Barnett C, Barnett Y (2004). An investigation of DNA excision repair in human CD4^+^ TCCs as a function of age *in vitro*. Exp Gerontol.

[6] Annett K, Duggan O, Freeburn F, Hyland P, Pawelec G, Barnett Y (2005). An investigation of DNA mismatch repair capacity under normal culture conditions and under conditions of supraphysiological challenge in human CD4^+^ TCCs from donors of different ages. Exp Gerontol.

[7] Barnett YA, Barnett CR (1998). DNA damage and mutation: contributors to the age related alterations in T cell-mediated immune responses?. Mech Ageing Dev.

[8] Hyland P, Barnett C, Pawelec G, Barnett Y (2001). Age-related accumulation of oxidative DNA damage and alterations in levels of p16, p21 and p27 in human CD4^+^ TCCs *in vitro*. Mech Ageing Dev.

[9] Barnett YA, King C, Bristow-Craig H, Warnock C, Hyland P, Gillespie E, Rea M, Middleton D, Curren M, Pawelec G, Barnett CR, Pawelec G (1999). Age related increases in DNA damage and mutations in T cells in vivo and in vitro: contributors to alterations in T cell mediated immune responses?. EUCAMBIS: Immunology and Ageing in Europe.

[10] Marthandan SS, Hyland P, Pawelec G, Barnett YA (2013). An investigation of the effects of the antioxidants, Ebselen or N-acetyl cysteine on human peripheral blood mononuclear cells and T cells. Immun Ageing.

[11] Barnett YA, King CM (1995). An investigation of in vivo antioxidant status, mutation and DNA repair capacity in human lymphocytes as a function of age. Mutat Res.

[12] Azad GK, Tomar RS (2014). Ebselen, a promising antioxidant drug: mechanisms of action and targets of biological pathways. Mol Biol Rep.

[13] Lasram MM, Lamine AJ, Dhouib IB, Bouzid K, Annabi A, Belhadjhmida N, Ahmed MB, El Fazaa S, Abdelmoula J, Gharbi N (2014). Antioxidant and ant-inflammatory effects of N-acetyl cysteine against malathion-induced liver damages and immunotoxicity in rats. Life Sci.

[14] Ligthart GJ, Corberand JX, Goertzen HGM, Minders AE, Knook DL, Hijmans W (1990). Necessity of the assessment of health status in human immunogerontological studies: evaluation of the SENIEUR protocol. Mech Ageing Dev.

[15] Pawelec G, Adibzadeh M, Rehbein A, Hähnel K, Wagner W, Engel A (2000). *In vitro* senescence models for human T lymphocytes. Vaccine.

[16] McCarron M, Osborne Y, Story C, Dempsey JL, Turner R, Morley A (1987). Effect of age on lymphocyte proliferation. Mech Ageing Dev.

[17] Wayne SJ, Rhyne RL, Garry PJ, Goodwin JS (1990). Cell mediated immunity as a predictor of morbidity and mortality in subjects over 60. J Gerontol.

[18] Ferguson FG, Wikby A, Maxson P, Olsson J, Johansson B (1995). Immune parameters in a longitudinal study of a very old population of Swedish people: a comparison between survivors and nonsurvivors. J Gerontol A Biol Sci Med Sci.

[19] Franceschi C, Monti D, Barbieri D, Grassilli E, Troiano L, Salvioli S, Negro P, Capri M, Guido M, Azzi R, Sansoni P, Paganelli R, Fagiolo U, Baggio G, Donazzan S, Mariotti S, D’addato S, Gaddi A, Ortolani C, Cossarizza A (1995). Immunosenescence in humans: deterioration or remodelling?. Int Rev Immunol.

[20] Yang CF, Shen HM, Ong CN (1999). Protective effect of ebselen against hydrogen peroxide induced cytotoxicity and DNA damage in HepG_2_ cells. Biochem Pharmacol.

[21] Zhang L, Zhou L, Du J, Li M, Qian C, Cheng Y, Peng Y, Xie J, Wang D: **Induction of apoptosis in human multiple myeloma cell lines by ebselen via enhancing the endogenous reactive oxygen species production.** *Biomed Res Int* 2014, doi:10.1155/2014/696107. 10.1155/2014/696107 PMC3921973 24587987

[22] Li J, Chen JJ, Zhang F, Zhang C (2000). Ebselen protection against H_2_O_2_ induced cytotoxicity and DNA damage in HL-60 cells. Acta Pharmacol Sin.

[23] Yoshizumi M, Kogame T, Suzaki Y, Fujita Y, Kyaw M, Kirima K, Ishizawa K, Tsuchiya K, Kagami S, Tamaki T (2002). Ebselen attenuates oxidative stress-induced apoptosis via the inhibition of the c-Jun N-terminal kinase and activator protein-1 signalling pathway in PC12 cells. Br J Pharmacol.

[24] Han D, Sen CK, Roy S, Kobayashi MS, Tritschler HJ, Packer L (1997). Protection against glutamate induced cytotoxicity in C6 glial cells by thiol antioxidants. Am J Physiol Regul Integr Comp Physiol.

[25] Wagner R, Heckman HM, Myers RR (1998). Wallerian degeneration and hyperalgesia after peripheral nerve injury are glutathione dependent. Pain.

[26] Hansen JM, Watson WH, Jones DP (2004). Compartmentation of Nrf-2 redox control. Regulation of cytoplasmic activation by glutathione and DNA binding by thioredoxin-1. Toxicol Sci.

[27] Kamata H, Hirata H (1999). Redox regulation of cellular signalling. Cell Signal.

[28] Pani G, Colavitti R, Borello S, Galeotti T (2000). Endogenous oxygen radicals modulate protein tyrosine phosphorylation and JNK-1 activation in lectin stimulated thymocytes. J Biochem.

[29] Zou T, Yang W, Hou Z, Yang J (2010). Homocysteine enhances cell proliferation in vascular smooth muscle cells: role of p38 MAPK and p47phox. Acta Biochim Biophys Sin (Shanghai).

[30] Meshkini A, Yazdanparast R (2010). Involvement of oxidative stress in taxol induced apoptosis in chronic myelogenous leukemia K562 cells. Exp Toxicol Pathol.

[31] Pawelec G, Barnett Y, Forsey R, Frasca D, Globerson A, McLeod J, Caruso C, Franceschi C, Fulop T, Gupta S, Mariani E, Mocchegiani E, Solana R (2002). T cells and ageing. Front Biosci.

[32] Sivakumar S, Daum JR, Tipton AR, Rankin S, Gorbsky GJ (2014). The spindle and kinetochore associated (Ska) complex enhances binding of the anaphase promoting complex/cyclosome (APC/C) to chromosomes and promote s mitotic exit. Mol Biol Cell.

[33] Grubeck- Leobenstein B, Lechner H, Trieb K (1994). Long term in vitro growth of human TCCs: can post-mitotic senescent cell populations be defined?. Int Arch Allergy Immunol.

[34] Morgenstern R, Cotgreave IA, Engman L (1992). Determinations of the relative contributions of the diselenide and selenol forms of ebselen in the mechanism of its GPx like activity. Chem Biol Interact.

[35] Yang CF, Shen HM, Ong CN (2000). Ebselen induces apoptosis in HepG_2_ cells through rapid depletion of intracellular thiols. Arch Biochem Biophys.

[36] Maher P (2005). The effect of stress and ageing on glutathione metabolism. Ageing Res Rev.

[37] Hall AG (1999). The role of glutathione in the regulation of apoptosis. Eur J Clin Invest.

[38] Ghibelli L, Rotilio CFG, Lafavia E, Coppola S, Colussi C, Civitareale P, Ciriola R (1998). Rescue of cells from apoptosis by inhibition of active GSH extrusion. FASEB J.

[39] Schafer FQ, Buettner GR (2001). Redox environment of the cell as viewed through the redox state of the glutathione disulfide/glutathione couple. Free Radic Biol Med.

[40] Pastore A, Piemonte F, Locatelli M, Lo Russo A, Gaeta LM, Tozzi G, Federici G (2001). Determination of blood total, reduced and oxidised glutathione in pediatric subjects. Clin Chem.

[41] Schreck R, Rieber P, Baeuerle PA (1992). Reactive oxygen intermediattes as apparently widely used messengers in the activation of the NF-kappa B transcription factor and HIV-1. EMBO J.

[42] Tatla S, Woodhead V, Foreman JC, Chain BM (1998). The role of ROS in triggering proliferation and IL-2 secretion in T cells. Free Radic Biol Med.

[43] Angel P, Karin M (1991). The role of Jun, Fos and the AP-1 complex in cell-proliferation and transformation. Biochim Biophys Acta.

[44] Zhang F, Lau SS, Monks TJ (2011). The cytoprotective effect of NAC against ROS induced cytotoxicity is independent of its ability to enhance glutathione synthesis. Toxicol Sci.

