# Supplementary material for: SENIEUR status of the originating cell donor negates certain ‘anti-immunosenescence’ effects of ebselen and N-acetyl cysteine in human T cell clone cultures
Source: Immun Ageing. 2014 Nov 30;11:17. doi: 10.1186/s12979-014-0017-5 (PMC4263119; doi:10.1186/s12979-014-0017-5)
Supplement: Additional file 1: Table S1. — Decrease in GSH:GSSG ratio levels with time in culture of 80 year old TCCs. Table S2. (A) Higher levels of oxidative DNA damage in 80 year old TCCs + / - Ebselen. Table S2. (B) Higher levels of oxidative DNA damage in 80 year old TCCs + / - NAC. Figure S1. (A-D) Effect of Ebselen on MAP kinase pathways in 26 year old TCCs. Figure S1. (E-H) Effect of Ebselen on MAP kinase pathways in 45 year old TCCs. Figure S1. (I-L) Effect of Ebselen on MAP kinase pathways in 80 year old TCCs. Figure S2. (A-D) Effect of NAC on MAP kinase pathways in 26 year old TCCs. Figure S2. (E-H) Effect of NAC on MAP kinase pathways in 45 year old TCCs. Figure S2. (I-L) Effect of NAC on MAP kinase pathways in 80 year old TCCs. Figure S3. (C) Impact of 30 μM Ebselen (C) on total levels of JNK, c-Jun, p38 and ERK. Figure S3. (D) Impact of 7.5 mM NAC (D) on total levels of JNK, c-Jun, p38 and ERK. [file 12979_2014_17_MOESM1_ESM.docx]

**Supplement Section**

**Table S1 Decrease in GSH:GSSG ratio levels with time in culture of 80 year old TCCs**

(A and B) The decrease in levels of GSH:GSSG ratio in CD4^+^ TCCs derived from an 80 year old donor with age when supplemented + / - 30 μM Ebselen (A) or 7.5 mM NAC (B) . * Significantly lower GSH:GSSG ratio in week 10 in comparison with weeks 2 and 5. n=3 pooled TCCs for each age group


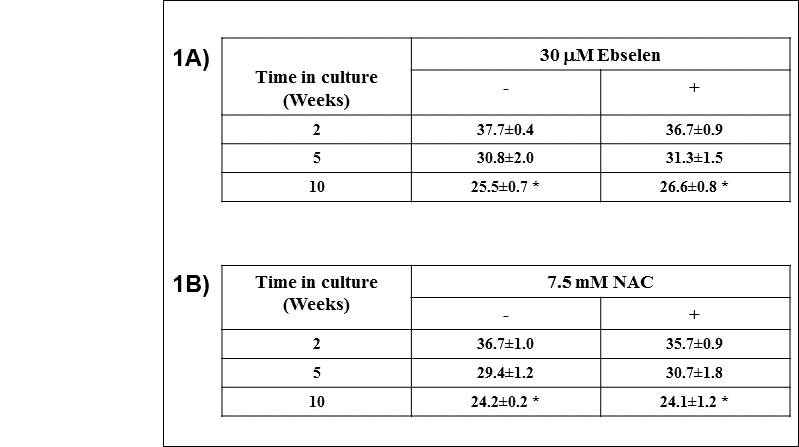


**Table S2 (A) Higher levels of oxidative DNA damage in 80 year old TCCs + / - Ebselen**

(A) The levels of oxidative DNA damage in three pooled CD4^+^ TCCs (+ / - 30 μM Ebselen) derived from an 80 year old donor was significantly higher than the levels in three pooled TCCs, derived from either 26 or 45 year old donor (both published data from [10]). * Significantly higher levels of oxidative DNA damage in 399-37 clones (80 year old) compared to both 400-23 (26 year old) and 385-7 (45 year old) clones.

**
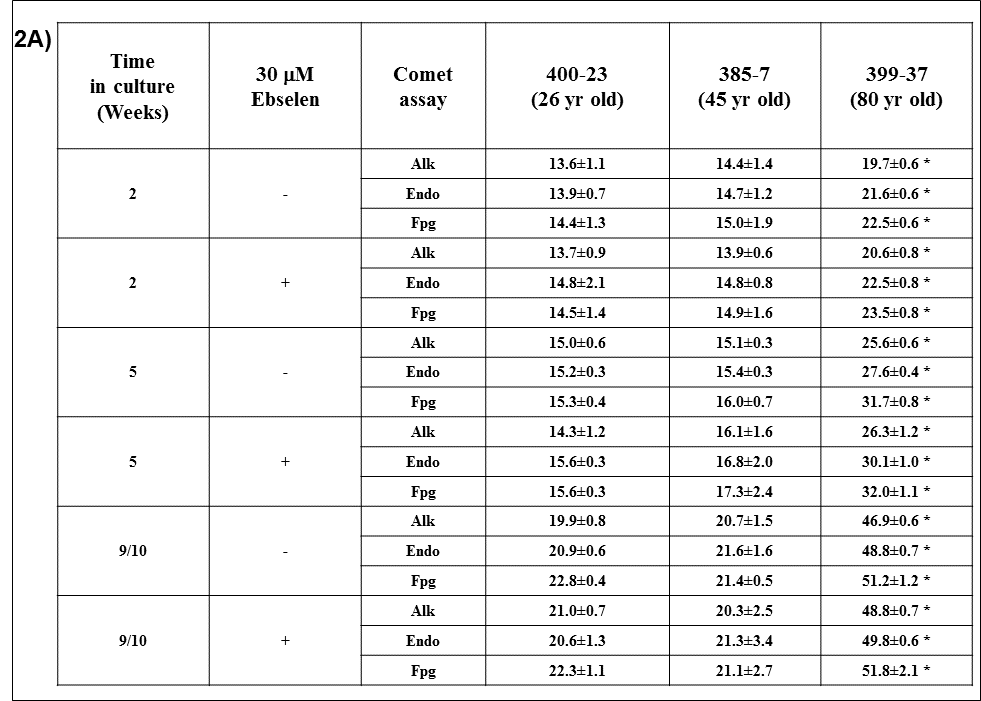
**

**Table S2 (B) Higher levels of oxidative DNA damage in 80 year old TCCs + / - NAC**

(B) The levels of oxidative DNA damage in three pooled CD4^+^ TCCs (+ / - 7.5 mM NAC) derived from an 80 year old donor was significantly higher than the levels in three pooled TCCs, derived from either 26 or 45 year old donor (both published data from [10]). * Significantly higher levels of oxidative DNA damage in 399-37 clones (80 year old) compared to both 400-23 (26 year old) and 385-7 (45 year old) clones.

**
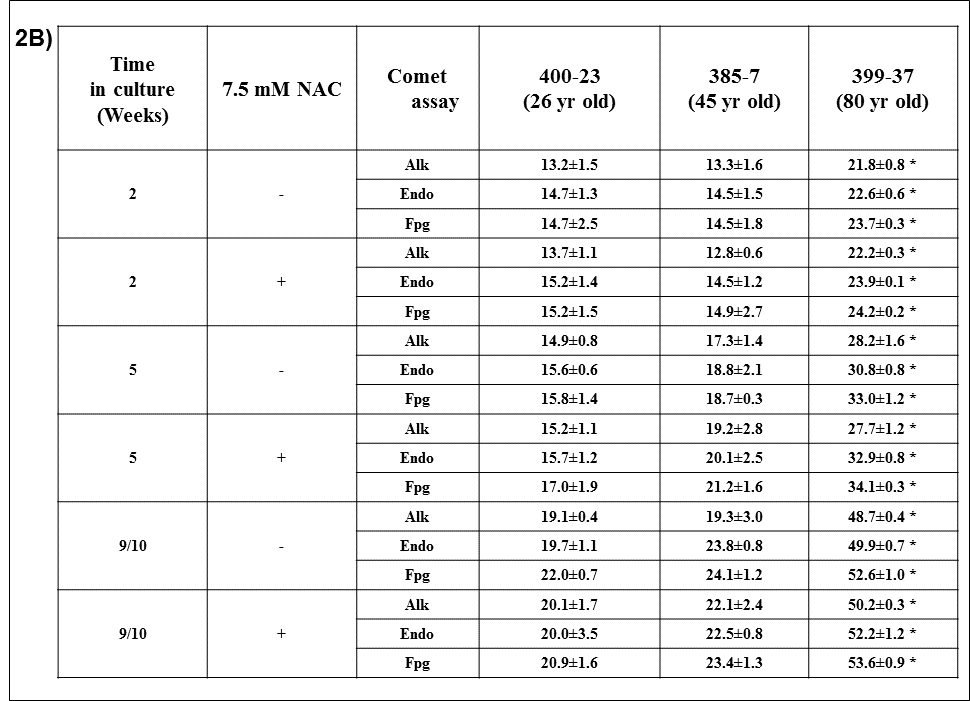
**


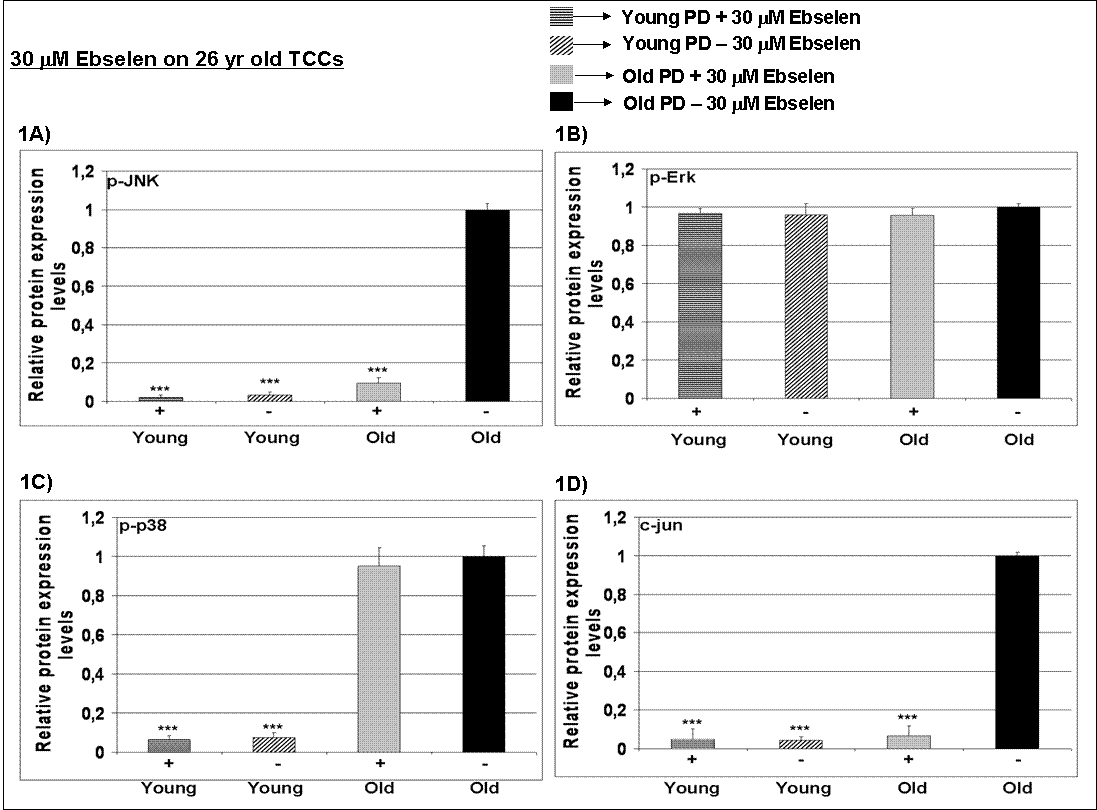


**Figure S1 (A-D) Effect of Ebselen on MAP kinase pathways in 26 year old TCCs**

Comparison of mean fold change of protein expression levels of p-JNK (A), p-ERK (B), p-p38 (C) and c-Jun (D) in early PD (young) and late PD (old) TCCs (derived from a 26 yr old donor) supplemented with 30 μM Ebselen compared to controls. In all the above figures, the intensity of the bands of old PD TCCs (- Ebselen treatment) were assigned 1. Samples were analysed in triplicate. The bars indicate the mean ± S.D. Young PD TCCs (+ / - Ebselen) and old PD TCCs (+ Ebselen) were compared to old PD TCCs (- Ebselen) using Student’s t-test and values statistically different to old PD TCCs (- Ebselen) are indicated with an asterix: *** p < 0.001 - significantly different compared to old TCCs (- Ebselen) with PD assigned 1.


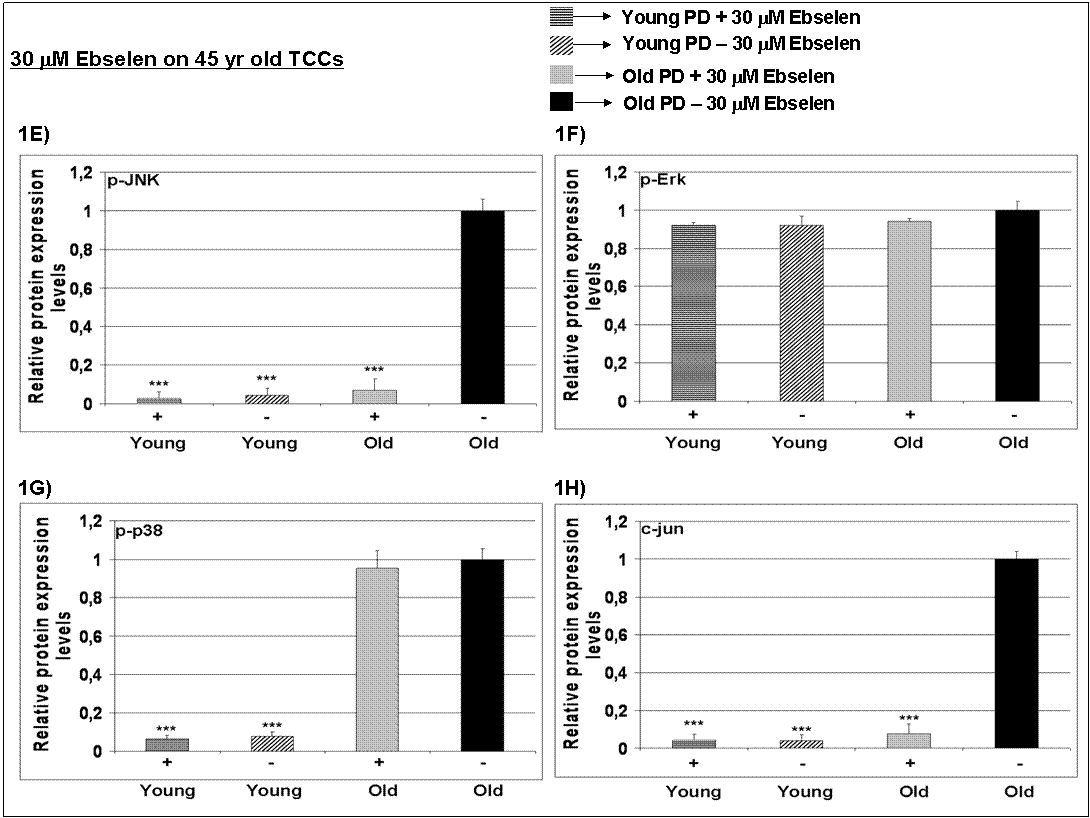


**Figure S1 (E-H) Effect of Ebselen on MAP kinase pathways in 45 year old TCCs**

Comparison of mean fold change of protein expression levels of p-JNK (E), p-ERK (F), p-p38 (G) and c-Jun (H) in early PD (young) and late PD (old) TCCs (derived from a 45 yr old donor) supplemented with 30 μM Ebselen compared to controls. In all the above figures, the intensity of the bands of old PD TCCs (- Ebselen treatment) were assigned 1. Samples were analysed in triplicate. The bars indicate the mean ± S.D. Young PD TCCs (+ / - Ebselen) and old PD TCCs (+ Ebselen) were compared to old PD TCCs (- Ebselen) using Student’s t-test and values statistically different to old PD TCCs (- Ebselen) are indicated with an asterix: *** p < 0.001 - significantly different compared to old TCCs (- Ebselen) with PD assigned 1.

**
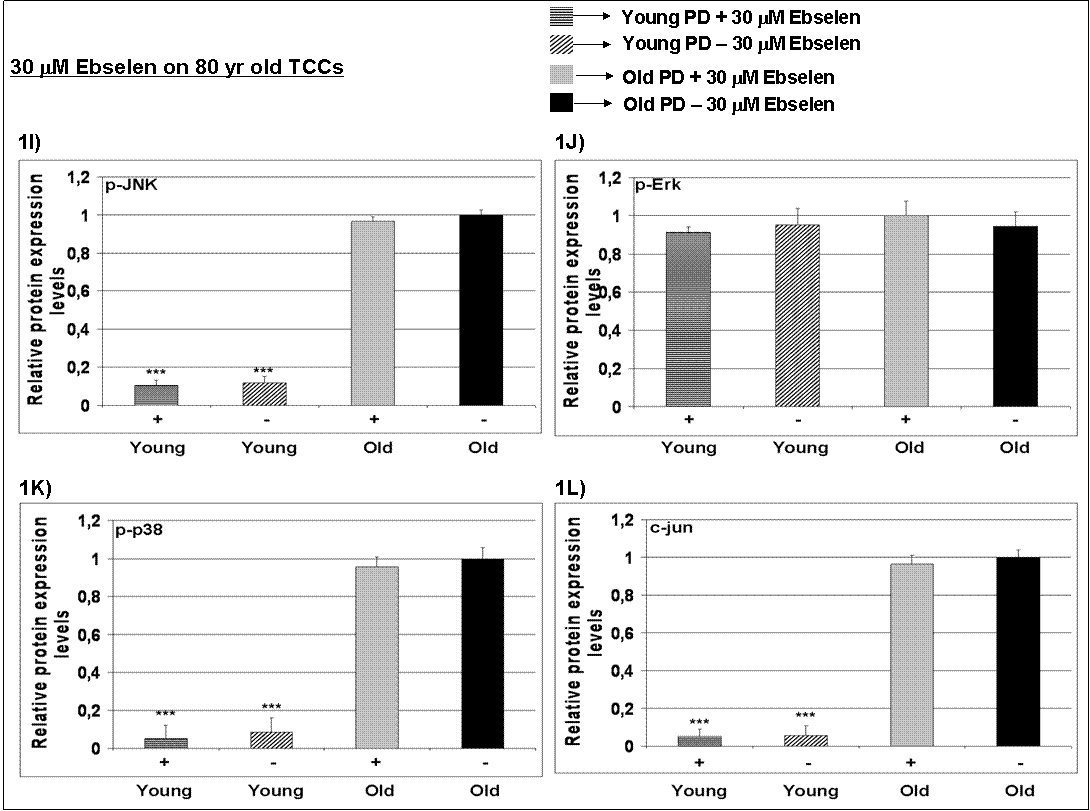
**

**Figure S1 (I-L) Effect of Ebselen on MAP kinase pathways in 80 year old TCCs**

Comparison of mean fold change of protein expression levels of p-JNK (I), p-ERK (J), p-p38 (K) and c-Jun (L) in early PD (young) and late PD (old) TCCs (derived from a 80 yr old donor) supplemented with 30 μM Ebselen compared to controls. In all the above figures, the intensity of the bands of old PD TCCs (- Ebselen treatment) were assigned 1 whereas in figure 1J, intensity of the bands of old PD TCCs (+ Ebselen treatment) was assigned 1. Samples were analysed in triplicate. The bars indicate the mean ± S.D. Young PD TCCs (+ / - Ebselen) and old PD TCCs (+ Ebselen) were compared to old PD TCCs (- Ebselen) using Student’s t-test except for figure 1J where old PD TCCs (+ Ebselen) was assigned as the template to be compared. The values statistically different to old PD TCCs (- Ebselen) with the exception of figure 1J are indicated with an asterix: *** p < 0.001 - significantly different compared to old TCCs (- Ebselen) with PD assigned 1 except for figure 1J where old PD TCCs (+ Ebselen) was assigned the value 1.


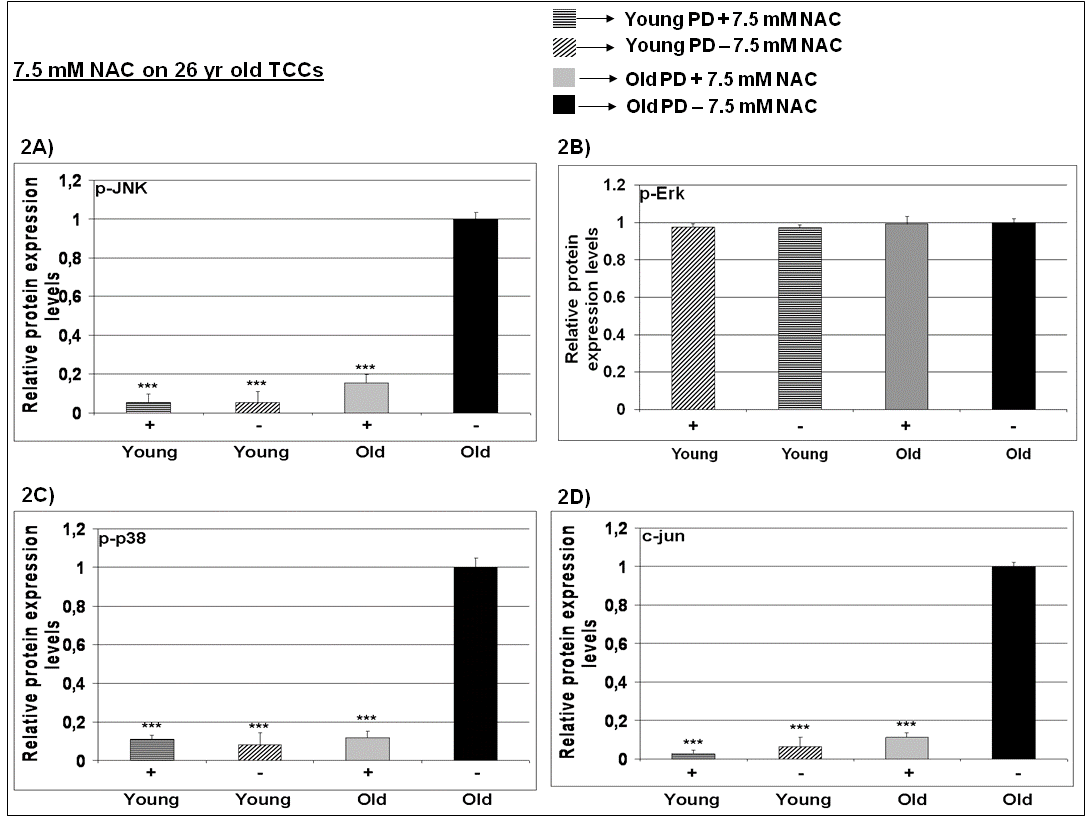


**Figure S2 (A-D) Effect of NAC on MAP kinase pathways in 26 year old TCCs**

Comparison of mean fold change of protein expression levels of p-JNK (A), p-ERK (B), p-p38 (C) and c-Jun (D) in early PD (young) and late PD (old) TCCs (derived from a 26 yr old donor) supplemented with 7.5 mM NAC compared to controls. In all the above figures, the intensity of the bands of old PD TCCs (- NAC treatment) were assigned 1. Samples were analysed in triplicate. The bars indicate the mean ± S.D. Young PD TCCs (+ / - NAC) and old PD TCCs (+ NAC) were compared to old PD TCCs (- NAC) using Student’s t-test and values statistically different to old PD TCCs (- NAC) are indicated with an asterix: *** p < 0.001 - significantly different compared to old TCCs (- NAC) with PD assigned 1.


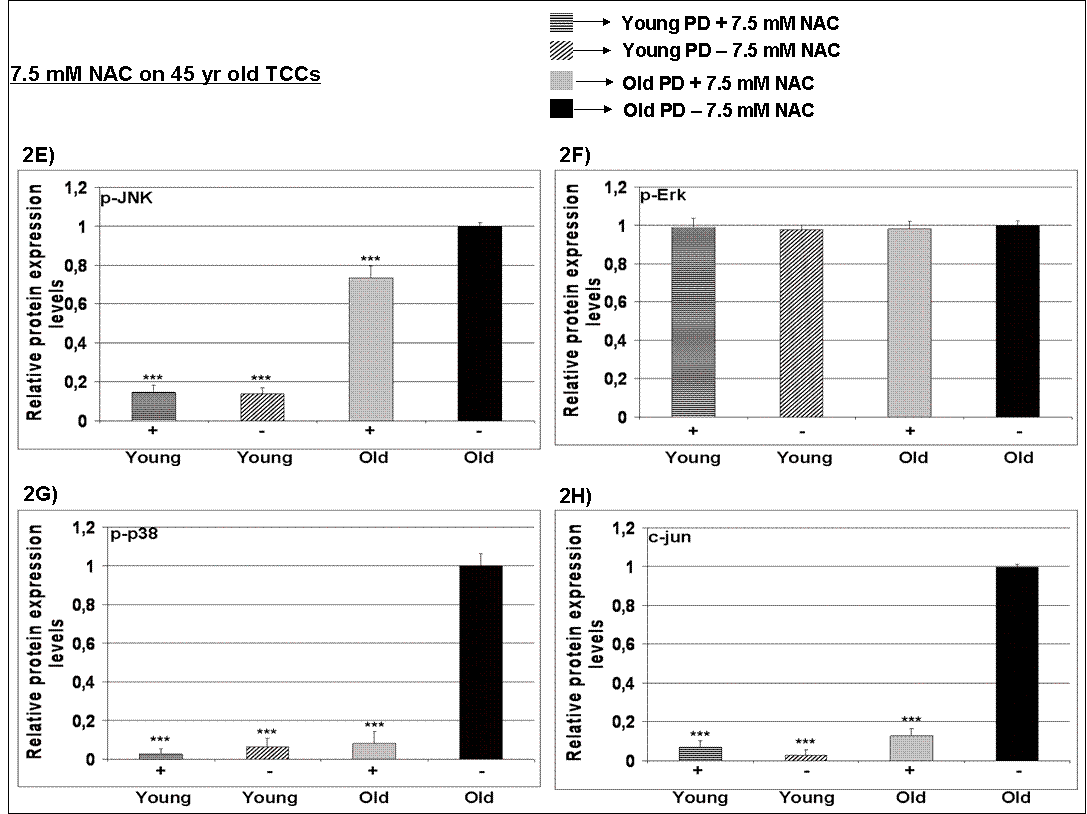


**Figure S2 (E-H) Effect of NAC on MAP kinase pathways in 45 year old TCCs**

Comparison of mean fold change of protein expression levels of p-JNK (E), p-ERK (F), p-p38 (G) and c-Jun (H) in early PD (young) and late PD (old) TCCs (derived from a 45 yr old donor) supplemented with 7.5 mM NAC compared to controls. In all the above figures, the intensity of the bands of old PD TCCs (- NAC treatment) were assigned 1. Samples were analysed in triplicate. The bars indicate the mean ± S.D. Young PD TCCs (+ / - NAC) and old PD TCCs (+ NAC) were compared to old PD TCCs (- NAC) using Student’s t-test and values statistically different to old PD TCCs (- NAC) are indicated with an asterix: *** p < 0.001 - significantly different compared to old TCCs (- NAC) with PD assigned 1.


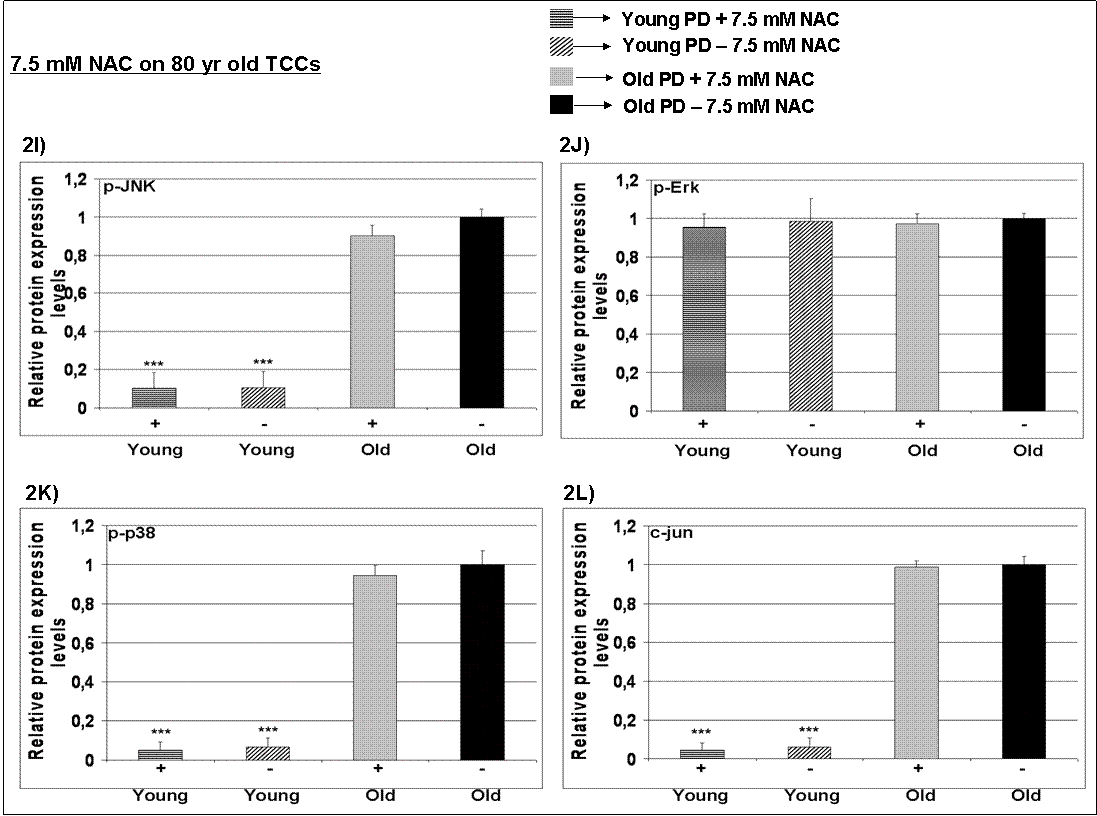


**Figure S2 (I-L) Effect of NAC on MAP kinase pathways in 80 year old TCCs**

Comparison of mean fold change of protein expression levels of p-JNK (I), p-ERK (J), p-p38 (K) and c-Jun (L) in early PD (young) and late PD (old) TCCs (derived from a 80 yr old donor) supplemented with 7.5 mM NAC compared to controls. In all the above figures, the intensity of the bands of old PD TCCs (- NAC treatment) were assigned 1. Samples were analysed in triplicate. The bars indicate the mean ± S.D. Young PD TCCs (+ / - NAC) and old PD TCCs (+ NAC) were compared to old PD TCCs (- NAC) using Student’s t-test and values statistically different to old PD TCCs (- NAC) are indicated with an asterix: *** p < 0.001 - significantly different compared to old TCCs (- NAC) with PD assigned 1.


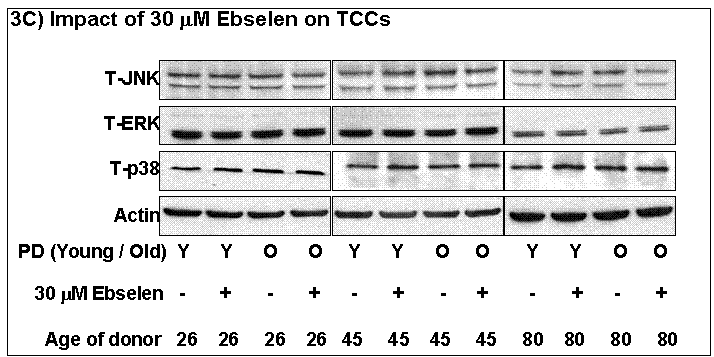


**Figure S3 (C)** Impact of 30 μM Ebselen (C) on total levels of JNK, c-Jun, p38 and ERK. The blots reveal the effect of antioxidant supplementation between the young (early PD) and aged (late PD) TCCs isolated from healthy 26, 45 or 80 year old donors compared to non-supplemented controls


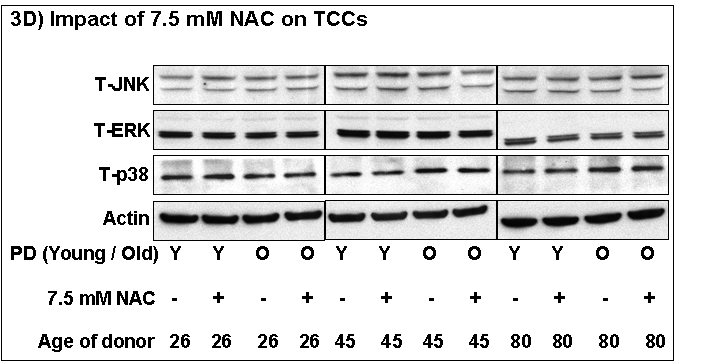


**Figure S3 (D)** Impact of 7.5 mM NAC (D) on total levels of JNK, c-Jun, p38 and ERK. The blots reveal the effect of antioxidant supplementation between the young (early PD) and aged (late PD) TCCs isolated from healthy 26, 45 or 80 year old donors compared to non-supplemented controls
